# Electrochemical Sensors Based on Carbon Nanotubes

**DOI:** 10.3390/s90402289

**Published:** 2009-03-30

**Authors:** A. J. Saleh Ahammad, Jae-Joon Lee, Md. Aminur Rahman

**Affiliations:** 1 Department of Advanced Technology Fusion, Konkuk University, Seoul 143-701, Korea; 2 Department of Applied Chemistry, Konkuk University, Chungju 380-701, Korea

**Keywords:** Carbon Nanotubes, Modified Electrodes, Electrochemical Sensors, Biosensors, Immunosensors, DNA sensors

## Abstract

This review focuses on recent contributions in the development of the electrochemical sensors based on carbon nanotubes (CNTs). CNTs have unique mechanical and electronic properties, combined with chemical stability, and behave electrically as a metal or semiconductor, depending on their structure. For sensing applications, CNTs have many advantages such as small size with larger surface area, excellent electron transfer promoting ability when used as electrodes modifier in electrochemical reactions, and easy protein immobilization with retention of its activity for potential biosensors. CNTs play an important role in the performance of electrochemical biosensors, immunosensors, and DNA biosensors. Various methods have been developed for the design of sensors using CNTs in recent years. Herein we summarize the applications of CNTs in the construction of electrochemical sensors and biosensors along with other nanomaterials and conducting polymers.

## 1. Introduction

A sensor is a device which detects a variable quantity, usually electronically, and converts the measurement into signals to be recorded elsewhere. The most important aspect of investigation of sensors is sensitivity, selectivity, and stability. Sensors can be classified, according to the type of energy transfer, as thermal, electromagnetic, mechanical, and electrochemical. Among them, the electrochemical sensors are very promising analytical methods because of their high degree of selectivity and sensitivity. They are more useful and easy to determine the concentrations of various analytes in samples such as fluids and dissolved solid materials. They are frequently used in clinical diagnostics, occupational safety, medical engineering, process measuring engineering, and environmental analysis.

Currently, much attention has been focused on developing nanomaterials, which are used for signal amplification in electrochemical sensors. Nanomaterials are usually used to take advantage of a larger surface area for biomolecules to be immobilized. This generally increases the number of binding sites available for the detection of a specific chemical analyte [1]. Various types of nanomaterials are used in electrochemical sensors. Carbon nanotubes (CNTs) [2] are one of the most exciting materials because of their unique electronic, chemical, and mechanical properties [3]. CNTs possessed sp^2^ carbon units with several nanometers in diameter and many microns in length. Two groups of CNTs, multi-walled (MW) and single-walled (SW), can be synthesized by electrical arc discharge, laser vaporization, and chemical vapor deposition methods. CNTs behave as either metals or semiconductors, depending on the diameter and the degree of helicity [4]. They are suitable for the modification of various electrodes due to their high electronic conductivity for the electron transfer reactions and better electrochemical and chemical stabilities in both aqueous and non-aqueous solutions [5]. Furthermore, construction of efficient electrochemical sensors using the CNTs-modified electrodes is very promising in that they promote electron-transfer reactions in several small biologically important molecules and large biomolecules [6,7].

This review focuses on the use of CNTs for electrochemical sensors and biosensors. Thousands of paper have been published in this field during last decade and therefore the references that appeared before 2006 were not included in this manuscript. Electrochemical detection based on a voltammetric and chronopotentiometric techniques was mostly discussed, whereas some other detection techniques related with electrophoresis, chromatography, and lab-on-a-chip were not included.

## 2. Electrochemistry of Carbon Nanotubes

**Figure 1.**
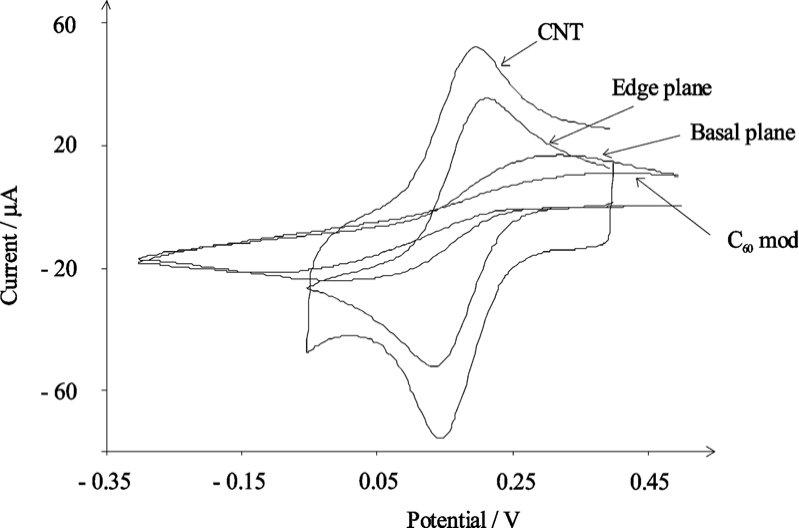
Cyclic voltammograms for the reduction of 1 mM ferricyanide for different electrodes at a scan rate of 100 mV s^−1^ [10]. Reproduced by permission of The Royal Society of Chemistry.

CNTs are electrochemically inert materials similar to other carbon-based materials used in electrochemistry, i.e. glassy carbon, graphite, and diamond. They possess distinct electrochemical properties because of their unique electronic structure. The carbon atoms of CNTs at the sidewall and the end of the tubes are not same and their behavior can be compared with the basal plane and edge plane of highly oriented pyrolytic graphite (HOPG), respectively [8]. Compton *et al*. studied the redox reaction of ferricyanide at the C_60_- and nanotube modified electrodes and compared these results with basal and edge planes pyrolytic graphite electrodes. They observed similar electron transfer rate constants for CNTs-modified and the edge plane HOPG electrodes. They reported that the CNTs acted as an efficient electron transfer promoter [Figure 1] [9–11]. To illustrate the electron transfer kinetics, Holloway *et al*. studied the voltammetry of two standard redox processes of Fe(CN)_6_^4−^ and Ru(NH_3_)_6_^3+^ using three types of MWCNTs having oxygenated edge-plane, edge-plane, and almost no edge-plane like defects [12]. The rate of electron transfer was determined to be faster in the case of edge-plane defect sites. This further indicates that the electroactive sites on MWCNTs are located at the tube ends. The electrochemical properties of ferricyanide redox couple at aligned and randomly dispersed SWNTs-modified electrodes was studied by Gooding *et al*. [13,14]. For an acid-treated aligned SWNTs-modified electrode, a peak separation of 59 mV with a half-wave potential (E_1/2_) of 0.231 V (*vs*. Ag/AgCl) for the ferricyanide redox couple was observed. On the other hand, the peak separation was observed to be 99 mV when the acid-treated but randomly dispersed SWNTs modified electrode was used. The aligned SWNTs modified electrode shows the better electrochemical properties. It means that the electrochemistry of CNTs is dominated by the ends of the tubes.

The length of the aligned CNTs also has a significant effect on the electron transfer rate. The electron transfer rate constant varied inversely with the mean length of the CNTs. Gooding *et al*. investigated the effect of the length of CNTs on the apparent electron transfer rate constant of the surface attached ferrocenemethylamine on to the vertically aligned CNTs with cutting times of 2, 4, and 6 hrs during acid treatment [15]. The apparent rate constants were determined to be 98 ± 25, 187 ± 98, and 459 ± 132 s^−1^ for the mean lengths of 1175, 507, and 257 nm, respectively. When the nanotube dispersed randomly, the rate constant was found to be 12 ± 8 s^−1^ for a mean tube length of 257 nm, which was 40 times slower than that obtained at vertically aligned nanotubes modified electrode. The electron transfer rate at the CNTs modified electrode could be further increased by dialyzing the CNTs after purification and shortening treatment [16]. During purification and shortening of the SWNTs in concentrated nitric and sulfuric acids mixture, some residual acid moieties adsorbed on single-walled carbon nanotubes (SWNTs). Using TEM and HR-TEM techniques, Pumera *et al*. confirmed that CNTs contain residual metal impurities after acid wash with concentrated nitric acid at temperature of 80 °C [17]. These acid moieties can be reduced by dialyzing the purified and dialyzed CNTs against a solution of Triton^®^ X-100. The electrochemical measurements using self-assembled ferrocene-functionalized nanotube monolayers on a gold electrode showed that the dialyzed nanotubes exhibited a faster rate of electron transfer compared to the nondialyzed nanotubes [16].

The adsorbed acid moieties during purification and acid-treatment processes can also decrease the electrocatalytic activity of CNTs in electroanalysis. Pumera and his colleague suggested the use of dc magnetic susceptibility and electron paramagnetic resonance for screening and quality control of CNTs before use them in electroanalysis [18]. Recently, Dai and co-workers reported that vertically aligned nitrogen-doped CNTs can act as a metal-free electrode with a much better electrocatalytic activity [19]. The electrocatalytic activity and the electroanalytical performance at CNTs modified electrodes are strongly depended on the mode of production of the CNTs, either by chemical vapor deposition (CVD) or the ARC discharge process [20]. CNTs produced by CVD appear to be more electrochemically reactive in their voltammetric study than those produced using the ARC methodology. The differences in the electrochemical reactivity are attributed to the smaller fraction of exposed edge planes at ARC-CNTs and higher density of edge plane defects at CVD-CNTs. The electrocatalytic activity of ARC-CNTs can be increased after pre-anodization. Wang’s group illustrated the effect of the electrochemical pretreatment of ARC- and CVD-prepared multi-walled CNTs using nicotinamide adenine dinucleotide, ascorbic acid, hydrazine, and hydrogen peroxide model redox systems [21]. The fact that the ARC-CNTs display a marked improvement in their electrochemical reactivity, which indicates that the pre-anodization effectively breaks the end caps of ARC-CNTs to expose new edge plane-like sites.

## 3. Carbon Nanotube-Based Electrochemical Sensors

There have been many reports on CNTs-based electrochemical sensors. Various electrochemical techniques were used for sensing of biomolecules. Some common techniques are voltammetry, amperometry, potentiometry, impedemetry, and conductometry, which have been described in our previous review paper [22]. Voltammetry, measuring the current as a response to the applied potential, is one of the most useful and widespread technique among them. For enhanced current response, it is very important to develop a stable and highly target specific interface by various surface modification of conventional electrodes. The sensitivity and the selectivity are the crucial issues for the development of sensors for detecting biologically important molecules. Much effort has been made in the development of a highly sensitive and selective method for the detection of dopamine (DA), which is one of the important catecholamine neurotransmitters in the mammalian central nervous system. Conventional electrodes are not suitable for the determination of DA due to the interference from ascorbic acid (AA) and uric acid (UA), which are co-existed in a real sample at 100 times higher concentration than DA. These compounds can be easily oxidized at the similar potential of DA and thus always interfere with DA detection. The CNTs-modified electrodes have been widely used to resolve this problem. Wang *et al*. reported the fabrication of a poly (3-methylthiophene) modified glassy carbon electrode (GCE) coated with Nafion/SWCNTs for highly selective and sensitive determination of DA [23]. The modified electrode enhanced the voltammetric signal of DA and effectively suppressed the interferences of AA and UA as well. A lower detection limit of 5.0 nM was achieved for DA. It was also successfully applied for the determination of DA in healthy human blood serum. A CNT-polymer composite-modified electrode, with poly(styrenesulfonic acid) sodium salt and SWCNTs, were used for selective detection of DA [24]. The negatively charged poly (styrene sulfonic acid) sodium salt attracted positively charged DA in pH 7 PBS and selectively detected it from the interference of AA.

Polymer-MWCNTs composite can be used for the fabrication of DA sensor. Yin *et al*. developed a DA sensor using β-cyclodextrin-incorporated MWCNTs on a polyaniline (PANI) modified GCE [25]. A superior transducing property of PANI, a rapid electron transfer capability of MWCNTs, and the preconcentration by β-cyclodextrin showed the excellent sensitivity, selectivity, stability, and reproducibility in the determination of DA. Recently, Angeles *et al*. also developed an amperometric sensor for DA detection by using β-cyclodextrin and MWCNTs without polymer [26]. The enhancement of the oxidation current of DA was possible due to its diffusion through the β-cyclo-dextrin cavities and the facile contact with the dispersed MWCNTs. Moreover, Li *et al*. found that polypyrrole-SWCNTs composite film can detect DA, AA, and UA simultaneously, and it showed the electrocatalytic activity towards the oxidation of nitrite [27]. Zhou’s group reported that the modified electrode with poly (acrylic acid) and MWCNTs can suppress the oxidation peak of AA but enhance the DA and UA signals [28]. The application of poly (neutral red) functionalized MWCNTs was also reported by Yogeswaran and Chen for the simultaneous detection of AA, UA, and DA [29]. The clear separation of peaks was attributed to the electrostatic and hydrophobic interaction between the three analytes and the fixed cationic sites on polymer backbone as well as the functionalized MWCNTs, which are negatively charged.

Another important catecholamine neurotransmitter is epinephrine (EP), which is involved in the message transfer of the mammalian central nervous system. In biological fluids such as blood and urine, EP coexists with AA, and UA, so AA and UA may interfere during the electrochemical detection of EP at an unmodified electrode. CNTs-modified electrodes have been successfully used for the determination of EP. Chen’s group developed a method for simultaneous determination of AA, EP, and UA at physiologically relevant conditions by using the composite film composed of functionalized-MWCNTs and Nafion incorporating platinum and gold nanoparticles [30]. The oxidation peaks for AA, EP, and UA were separately observed and thus, the detections of these compounds did not interfere with each other. An EP sensor prepared by an *in-situ* electropolymerization of brilliant cresol blue (BCB) was reported by Yi *et al*. [31]. In this work, the GCE modified by the film of polymeric BCB and functionalized MWCNTs composite were used to detect EP. A low detection limit of 10 nM was obtained by using the BCB and functionalized MWCNTs nanocomposite. However, the authors did not discuss the issue of interference from other biological compounds such as AA. Valentini *et al*. used functionalized SWCNTs instead of MWCNTs for the selective detection of EP in the presence of AA [32]. They used the stainless steel microelectrodes modified by hydroxyl group functionalized SWCNTs, which were deposited electrophoretically. The presence of electron-donating -OH groups on SWCNTs repels AA and attracts the positively charged EP. It provided a relatively high electrochemical sensitivity for EP up to the detection limit of 2.0 nM.

Nicotinamide adenine dinucleotide (NADH) is a coenzyme involved in a wide range of enzymatic reactions. The direct oxidation of NADH at a bare electrode needs a high overpotential. CNT-modified electrode can be used for the stable low potential detection of NADH. Zhai *et al*. developed a multilayer film of MWCNTs and chitosan (CS) using the layer-by-layer (LBL) method by taking advantage of the interaction between a positively charged CS and the negatively charged MWCNTs [33]. They assembled nine-layers of CS/MWCNTs successfully, which showed a very rapid and stable response of NADH oxidation at about 400 mV with the detection limit (S/N = 3) of 0.3 μM. A layer-by-layer approach is an efficient way to increase the amount of catalyst or enzyme at the sensor surface. However, the thickness of the multilayer need to be optimized as the sensor response can be suppressed by the very thick multilayer. Wang *et al*. fabricated an electrode by mixing a room-temperature ionic liquid, 1-butyl-3-methylimidazolium tetrafluoroborate, and MWCNTs along with CS. This electrode was used for NADH sensing with the detection limit of *ca.* 0.06 μM [34]. The use of ionic liquid and MWCNTs at the sensor surface may increase the surface ionic and electrical conductivities, thus, may enhance the sensitivity of the sensor. Radoi *et al*. used the covalently linked variamine blue, as a redox mediator to the oxidized SWCNTs for the detection of NADH [35]. The NADH oxidation potential was found to be lowered, from the changes of formal redox potential of the mediator, and therefore the sensor efficiency was improved due to the electrocatalytic activity of the mediator. Another very common approach for NADH sensors is the use of polymer composites with MWCNTs such as poly (acrylic acid)-wrapped MWCNTs complex [36], MWCNTs-poly (1,2-diaminobenzene) nanoporous composite [37], quinone-amine polymer- MWCNTs nanocomposite [38], and poly-(3-methylthiophene)-MWCNTs hybrid composite [39]. The sensitivities of the polymer-MWCNTs nanocomposite-based NADH detections were found to be enhanced due to the excellent electrocatalytic activities of the nanocomposites.

Hydrogen peroxide (H_2_O_2_) is a product of several biological, enzyme-catalyzed reactions. The detection of H_2_O_2_ plays an important role in food industry, environmental protection, and in medical diagnostics. For the sensitive detection of H_2_O_2_, Tkac and Ruzgas have used an electrode modified with SWCNTs. The sensitivity was highly dependent on the dispersing agent in the organic solvents and charging status of polymers (e.g. Nafion and CS) [40]. They found that the dispersion of both polymers is highly stable but the SWCNTs in the CS dispersion showed higher sensitivity for H_2_O_2_ compared to that in Nafion. Sun *et al*. introduced the modification with the ferrocene-filled SWCNTs for a H_2_O_2_ sensor with a good stability and reproducibility [41]. Ferrocene/ferrocenium (Fc/Fc^+^) was used as an electron-transfer mediator for the redox reaction of H_2_O_2_.

**Figure 2.**
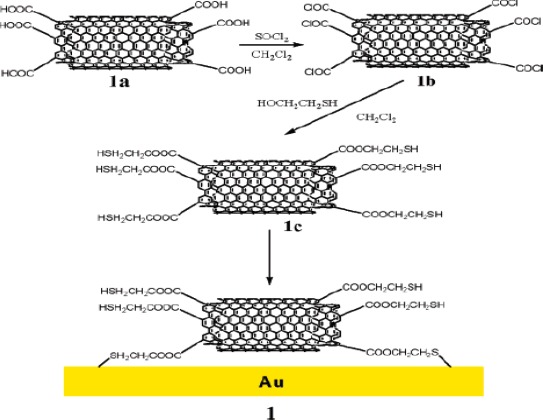
Scheme for the preparation of MWCNTs chemically modified electrode bearing SH groups. Reprinted with permission from [44]. Copyright (2006) American Chemical Society.

Electrochemical detections of metal ions have widely been studied using CNTs-modified electrode. For example, Yuan *et al*. developed a mercury-free electrode system by casting a dispersed solution of MWCNTs in Nafion on GCE [42]. It was applied for the detection of europium (III) by differential pulse adsorptive stripping voltammetry (DPASV) and a wide linear range from 40 nM to 10 mM with lower detection limit of 10 nM was obtained. Sun *et al*. used SWCNTs-Nafion film for the determination of Cd^2+^ in water [43]. Profumo *et al*. have prepared chemically modified MWCNTs electrode to detect the trace amount of As (III) and Bi (III) in a natural and high-salinity waters [44]. An oxidation of MWCNTs to introduce carboxyl groups and the subsequent chemical treatments were required to get a robust modification of the electrode surface for reliable measurement of saline water without desalting the sample (Figure 2).

Phosphate containing molecules such as phytic acid (PA), phosphomolybdic acid (PMA), dihexadecylphosphate (DHP), and dicetyl phosphate (DCP) have been widely used with CNTs for sensing applications. Jeon’s group recently developed an electrochemical sensor based on the modification of platinum electrode with SWCNTs and PA for the selective detection of DA in the presence of AA and UA [45]. The PA-SWCNTs films promoted the electron transfer reaction of DA while the PA in the films act as a binder and a negatively charged linker as well. The PA-SWCNTs/Pt electrode can separate the oxidation peak of DA from the interferences of electrochemical responses of AA and UA. Li *et al*. used PMA with MWCNTs to make an amperometric bromate sensor [46]. Hu and co-workers described a method for the preparation of a MWCNTs-DHP composite film on the GCE surface for the determination of lincomycin in tablets [47]. They found a well-defined oxidation peak for lincomycin by using cyclic voltammetry (CV) and a linear response range from 0.45 μM to 0.15 mM with the detection limit of 0.2 μM. The similar approach was applied by Ming *et al*. recently for the determination of trace Sudan I contamination in chili powder [48] and the same electrode was also used to determine the acyclovir voltammetrically [49]. DHP was replaced by DCP and 4-aminobenzenesulfonic acid (4-ABSA) for the determination of 2-chlorophenol [50] and tyrosine [51], respectively. The GCE modified with MWCNTs even without DHP was also used for the determination of procaine [52] and phenylephrine [53].

Rutin is a kind of flavonoid glycoside that has a wide range of physiological activities such as anti-inflammatory, antitumor, and antibacterial. CNT modified electrodes have been successfully using for the determination of rutin. A gold electrode modified with SWCNTs was fabricated, by Zeng *et al*., to investigate the voltammetric behavior of rutin [54]. At this electrode rutin exhibited an adsorption-controlled electrode reaction with a pair of peaks at ∼ 0.35V, implying two electron transfer process at the electrode surface. The linear range from 20 nM to 5.0 μM and the detection limit of 10 nM were determined. Yu’s group developed a rutin sensor based on GCE modified with β-cyclodextrin incorporated MWCNTs to take advantage of the inclusion interaction of β-cyclodextrin and rutin [55]. GCE modified by SWCNTs/poly (Neutral Red) composite film also exhibited a good catalytic activity on the redox properties of rutin [56]. Rutin, a kind of mediator was also used with MWCNTs for the fabrication of a hydroxylamine sensor [57].

Metal nanoparticles have received considerable attention in recent years. Incorporation of nanoparticles to CNTs for the modification of electrodes has been demonstrated to enhance the electrocatalytic activity of many electrochemical processes and therefore be suitable for sensing applications. Hrapovic *et al*. focused on metal nanoparticles/CNTs nanocomposites for electrochemical detection of trinitrotoluene (TNT) and other nitroaromatics [58]. They found that Cu nanoparticles and SWCNTs solubilized in Nafion provided the highest sensitivity for TNT with the detection limit of 1 ppb for analysis of TNT in tap water, river water, and contaminated soil. The composites of SWCNTs, gold nanoparticle (GNP), and ionic liquid (i.e. 1-octyl-3-methylimidazolium hexafluorophosphate) were used to fabricate a modified GCE for the sensitive voltammetric detection of chloramphenicol [59]. The composition of the film and the operation conditions played an important role in the voltammetric response and the detection limit under optimum condition was 5.0 nM. Electrochemically deposited Pt nano-clusters onto MWCNTs-modified GCE showed a strong electrocatalytic activity toward the oxidation of estrogens involving estradiol, estrone, and estriol [60]. This electrode showed the linear responses between 0.5–15 μM, 2.0–50 μM, and 1.0–75 μM for estradiol, estrone, and estriol, respectively, in the square-wave voltammetry. Wei and co-workers used composite of nano-silver coated MWCNTs to determine a trace of thiocyanate in urine and saliva samples from smokers and nonsmokers and observed the decrease of anodic current upon addition of trace amount of thiocyanate in nM level [61]. Liu *et al*. designed a sensor by electropolymerization of thionine at the GCE modified with GNPs/MWCNTs composites for simultaneous determination of adenine and guanine in DNA [62]. The detection limits (S/N = 3) of 10 nM and 8.0 nM were obtained for guanine and adenine, respectively.

Carbon-paste electrodes (CPEs) have been widely used as a suitable matrix for preparation of modified electrodes due to their simple preparation, renewability, and compatibility with various types of modifiers. CNT-modified CPEs showed considerable improvements in electrochemical behavior of materials. Zhuang *et al*. reported the fabrication of CPE modified with MWCNTs for the determination of bergenin using CV and differential pulse voltammetry (DPV) [63]. They found that the oxidation peak current of bergenin increased significantly upon increase of the contents of MWCNT and the linear dynamic range of 0.6 ∼ 10 μM was observed at an optimum experimental condition with a detection limit of 70 nM. Shahrokhian and Fotouhi added cobalt salophen (CoSal) for the determination of tryptophan [64]. The MWCNTs/CoSal CPE exhibited an electrocatalytic activity on the oxidation of tryptophan. Furthermore, this electrode could be used to detect tryptophan separately from the interferences of AA and cysteine. They also showed Nafion-incorporated MWCNTs/CoSal modified CPE can be used for the simultaneous detection of UA and AA. The detection limits were determined to be 60 and 100 nM, respectively [65]. When thionine-Nafion was incorporated into MWCNTs/CPE, it could be used to detect DA and AA simultaneously [66]. Recently, an electrode coated with SWCNTs paste was developed for sensitive voltammetric detection of methylparathion [67]. Fan *et al*. fabricated such electrode using ionic liquid (1-butyl-3-methyl-imidazolium hexafluophosphate) as a binder and they found a good electro-catalytic behavior to the electrochemical reduction of methylparathion. It was also found that the same electrode can be used to detect methylparathion and its hydrolysate (*p*-nitrophenol) simultaneously. The similar approach was applied for the voltammetric determination of xanthine [68] and UA [69]. A highly sensitive and fast responding electrochemical sensor was also prepared for piroxicam with MWCNTs paste electrodes by Abbaspour and Mirzajani [70]. It exhibited a high catalytic activity towards electrooxidation of piroxicam in a mild pH medium, showing the linear response range of 0.15–5 μg/mL, with the detection limit of 0.1 μg/mL. The same electrode was applied for the determinations of urapidil [71] and quercetin [72].

Composite film of CNTs with other materials such as conducting polymers, ceramics etc are very attractive combination of materials for the development of electrochemical sensors. Yang *et al*. fabricated a novel electrode using a poly(acid chrome blue K)/ MWCNTs composite film modified GCE for simultaneous detection of dihydroxybenzene isomers in real water samples by applying the first order linear sweep derivative voltammetry [73]. The detection limits for hydroquinone, catechol, and resorcinol were 100, 100, and 90 nM, respectively with this electrode. Wang *et al*. developed a novel sensor system by electrochemical oxidation of the mixture of l–cysteine and CNTs at GCE for the determination of terbinafine in human serum [74]. A composite film, found on GCE after oxidation, was responsible for the significant increase in the current response of terbinafine. It showed a wide range of linear response, 80 nM-50 μM, with the detection limit of 25 nM for terbinafine determination. Zhu *et al*. prepared CNTs ceramic composites electrode for electrochemical sensing by dispersing MWCNTs into the methyltrimethoxysilane derived sol–gel solution [75]. The electrode provided a better reversible behavior with a substantial decrease of overpotential, and a higher sensitivity compared to the unmodified one towards the electrochemical detection of ascorbic acid, uric acid, acetaminophenol, NADH, EP, DA, cysteine, and H_2_O_2_. Wang and coworkers have prepared a RuOx/CNTs-modified GCE by drop and dry process [76]. This electrode showed an electrocatalytic activity towards insulin and exhibited a wide range of linear response of 10 – 800 nM with a detection limit of 1 nM. Recently, Snider *et al*. developed a MWCNTs/dihydropyran composite film for the electrochemical detection of insulin secreted by islets in a microfluidic system [77]. Vega *et al*. developed a tetracycline antibiotic sensor based on MWCNTs modification of GCE [78]. A highly sensitive and reproducible electroanalytical response of tetracycline was attributed to the antifouling capability of the MWCNTs. Rezaei and Zare described a simple and highly sensitive method based on the modification of GCE by MWCNTs for the direct voltammetric determination of noscapine in pharmaceutical and clinical samples [79]. It significantly enhanced the noscapine signal and showed a wide linear range of 0.4 μM-10 mM, under the optimum condition, with the detection limit of 80 nM. They also applied the same electrode to study the determination of captopril with effective electrocatalysts, hexacyanoferrate (II) [80]. The system of MWCNTs and hexacyanoferrate strongly enhances the oxidation of captopril and a detection limit of 0.2 μM was obtained. Buratti *et al*. introduced cobalt oxide with the MWCNTs modified GCE for the detection of carbohydrates and thiols [81]. The modified electrode showed excellent electrocatalytic activity towards the oxidation of carbohydrates and thiols.

## 4. Carbon Nanotube-Based Electrochemical Biosensors

**Figure 3.**
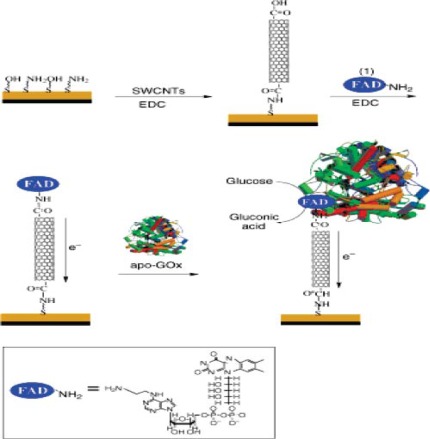
Assembly of the CNT electrically contacted glucose oxidase electrode. Reprinted with permission from [82]. Copyright (2008) American Chemical Society.

An electrochemical biosensor is an analytical tool for sensitive and selective detection of biomolecules. Increasing attention has been given to biosensors due to their potential applications in clinical chemistry, food industry, and environmental fields. Glucose oxidase (GOx)-based glucose biosensors are still considered as a primary model system in the development of new sensing materials and methods. They are the most extensively studied enzyme biosensors because of their high demand for blood glucose monitoring. A summary of recent progress in the field of glucose biosensors can be found in two excellent recent reviews by Wang and Heller [82,83]. CNTs are extremely attractive for fabricating electrochemical biosensors due to their outstanding properties, especially the excellent conductive, adsorptive and biocompatibility. Vertically aligned CNTs can be coupled with enzymes to provide a favorable surface orientation and act as an electrical connector between their redox center and the electrode surface [82]. Figure 3 showed the assembly of the CNT electrically contacted GOx electrode. Plugging enzymes into the CNTs by this way is an extremely efficient approach for the development of glucose biosensor.

Different types of glucose biosensors have been developed in recent years. Tsai and co-workers developed a nanobiocomposite film by incorporating functionalized MWCNTs and GOx into polypyrrole (PPy) film for a highly sensitive glucose biosensor [84]. The amperometric response of the optimized biosensor displayed a sensitivity of 95 nA/mM, a linear range up to 4 mM, and a response time of about 8 sec. Huang *et al*. loaded MWCNTs and GOx on a graphite disk using a LBL assembly technique to construct a glucose biosensor [85]. The current response to glucose was highly dependent on the number of layers and the maximum response was obtained at 6 layers of MWCNTs/GOx with the detection limit of 90 μM. Liu and Lin also applied LBL assembly technique to construct a sandwich-like structure, PDDA/GOx/PDDA/CNTs, for a reproducible and stable glucose biosensor while Zhao and Ju added poly (sodium 4-styrenesulfonate) with PDDA to construct multilayer membranes [86,87]. They modified gold electrode with 3-mercapto-1-propanesulfonic-acid and then bilayers of the PDDA and PSS were formed on the modified Au surface. PDDA wrapped MWCNTs and GOx was then assembled through LBL technique. Wang *et al*. functionalized gold electrodes with the negatively charged 11-mercaptoundecanoic acid (MUA) and then apply the LBL assembly of a positively charged redox polymer, poly [(vinylpyridine)Os(bipyridyl)_2_Cl^2+/3+^], and the negatively charged GOx/SWCNTs for glucose sensor. Liu *et al*. developed an amperometric glucose biosensor based on electrostatic assembly of GNPs/MWCNTs/GOx [88]. Positively charged poly (dimethyldi-allylammonium chloride) was used to connect them in a LBL pattern. The electrode showed an excellent electrocatalytic activity for glucose sensing at a relatively low potential (−0.2 V).

Xu *et al*. described an amperometric glucose biosensor based on an alternating electrostatic self-assembling GOx and dendrimer-encapsulated Pt nanoparticles (Pt-DENs) on MWCNTs [89]. The excellent electrocatalytic activity of CNTs and Pt-DENs toward H_2_O_2_ and special three-dimensional structure of the enzyme electrode resulted in a low detection limit with a wide linear response range, a high sensitivity with a good precision, and an enhanced operational stability. Shirsat *et al*. fabricated an amperometric glucose biosensor by applying a LBL assembly of SWCNTs and PPy multilayer film on a platinum coated with polyvinylidene fluoride (PVDF) membrane [90]. GOx was immobilized on the film by cross linking through glutaraldehyde (GA) (0.1%) and a linear response range from 1 mM to 50 mM of glucose concentration with the sensitivity of 7.06 uA/mM was achieved. A glucose sensor based on the LBL assembly of functionalized MWCNTs and poly (neutral red, PNR) multilayer film was also suggested [91]. This electrode showed a significant improvement of redox activity showing a synergic effect of excellent electron-transfer capability of CNTs and PNR. Another type of glucose biosensor was constructed by immobilizing GOx onto the electrode surface using GA. Yao and Shiu constructed a mediator type glucose sensor based on the immobilization of GOx at electropolymerized poly (toluidine blue O) film on CNTs modified GCE [92]. Poly (toluidine blue O) provided the polymer matrices to maintain the sensing activity and served as a redox mediator for enzymatic glucose oxidation. This biosensor showed enhanced current response at low potential (−0.1V) and therefore common interferences from AA, UA, and acetaminophen could be avoided. Zhu and co-workers suggested a Prussian Blue (PB) based amperometric glucose biosensor by assembling the PB nanoparticles on the surface of MWCNTs modified GCE followed by immobilization of GOx [93]. It showed good sensitivity, fast response with a detection limit of 12.7 mM. Similar PB based glucose biosensors were also prepared by immobilizing GOx in a film of LBL assembly of CS and MWCNTs [94] and on the nanocomposite film of PB nanoparticles/MWCNTs/poly (1,2-diamino-benzene) [95]. Manesh *et al*. fabricated a glucose biosensor based on the immobilization of GOx into an electrospun composite membrane consisting of polymethylmethacrylate (PMMA) dispersed with MWCNTs wrapped by a cationic PDDA polymer [96]. This nanofibrous electrode exhibited excellent electrocatalytic activity towards H_2_O_2_ with a pronounced oxidation current at +100 mV. Glucose was detected amperometrically with this nanofibrous electrode with a detection limit of 1 μM. A highly sensitive and selective glucose biosensor based on immobilization of GOx within mesoporous CNTs-titania-Nafion composite film coated on a platinized GCE, was also developed recently by Lee and co-workers [97]. It responded linearly to glucose in the wide range from 50 μM to 5.0 mM with sensitivity of 154 mA M^−1^cm^−2^.

A mediatorless glucose biosensor, based on the incorporation of GOx to the composite electrode of colloidal gold-CNTs-Teflon showed a remarkably higher sensitivity than that achieved with other GOx-CNTs bioelectrodes [98]. It could be used for ethanol biosensor by incorporating alcohol dehydrogenase (ADH) [99]. Chu *et al*. developed an amperometric glucose biosensor based on adsorption of GOx at the gold and platinum nanoparticles modified CNTs electrode where CNTs were covalently immobilized on cysteamine modified gold electrode [100]. The GOx/Au nano/Pt nano/CNTs/Au electrode was then covered with a thin layer of Nafion to avoid the loss of GOx and suppress the interfering signals from UA and AA. Recently, Zou *et al*. developed an amperometric glucose biosensor based on electrodeposition of platinum nanoparticles (PtNPs) onto MWCNTs and entrapping an enzyme in CS-SiO_2_ sol-gel [101]. This electrode showed an excellent electrocatalytic activity and high stability as well due to the synergistic action of Pt and MWCNTs and the biocompatibility of CS-SiO_2_ sol-gel. A wide linear range from 1 μM to 23 mM and a low detection limit of 1 μM was achieved for glucose sensing. Zhao *et al*. recently investigated an amperometric glucose biosensor based on PtNPs combined aligned CNTs electrode [102]. The combination of PtNPs and CNTs in this glucose biosensor showed a highly sensitive detection of glucose. Kang *et al*. constructed another glucose biosensor based on the integration of CNTs with gold-platinum alloy nanoparticles (Au-PtNPs) [103]. In this sensor, GOx was immobilized in biocompatible CS through cross-linking with GA on the Au–PtNPs/CNTs/CS film. It showed a low potential (0.1 V) detection of glucose with high sensitivity, low detection limit, good reproducibility, long-term stability, fast response, and high specificity. This biosensor was applied in the determination of glucose in real blood and urine samples with satisfactory results. Yang *et al*. prepared MWCNTs composite using Pt–NP doped sol/gel solution as a binder and incorporated GOx for glucose biosensor [104]. The sensitivity enhanced 4 times when Pt nanoparticles were loaded. A glucose biosensor, developed by Rivas and co-workers was based on the electrocatalytic activity of copper and iridium microparticles incorporated within the CNTs paste electrode containing GOx [105]. This biosensor detected glucose at very low potentials (−0.1 V) with high sensitivity and selectivity. Yao and Shiu examined the electrochemical and electrocatalytic properties of different types of CNTs material and used them for fabricating glucose biosensors [106]. They found that the electrodes modified with SWCNTs usually had better electron-transfer and electrocatalytic properties than the corresponding MWCNTs-modified electrodes. Recently, Jia *et al*. reported the fabrication of needle-type glucose biosensor by packing a mixture of MWCNTs, graphite powder, and freeze-dried GOx powder into a glass capillary of 0.5 mm inner diameter [107]. It showed an improved sensitivity and stability when the experimental condition was optimized. Zhu and co-workers proposed a bienzymatic mediatorless glucose biosensor based on co-immobilization of GOx and horseradish peroxidase (HRP) in an electropolymerized PPy film on a SWCNTs modified electrode [108]. They took advantage of direct electron transfer characteristics of HRP with CNTs electrode and realized a lower operational potential for selective determination with a minimized interference.

The detection of hydrogen peroxide (H_2_O_2_) is very important because many enzymatic biosensors rely on the detection of H_2_O_2_ generated by an enzymatic reaction. Since the amount of generated H_2_O_2_ from an enzymatic reaction is very low, the fabrication of a highly sensitive H_2_O_2_ biosensor is needed. CNTs can be used in the fabrication of highly sensitive H_2_O_2_ biosensors. There have been many reports on CNTs based H_2_O_2_ biosensors. Chen and Lu reported the encapsulation of hemoglobin (Hb) in the composite film of carboxylic acid functionalized MWCNTs and polyelectrolyte-surfactant polymer to develop a H_2_O_2_ biosensor [109]. Faradic response of the Hb was observed and it exhibited excellent electrocatalytic activity to reduce H_2_O_2_. Chen *et al*. proposed an amperometric third-generation H_2_O_2_ biosensor based on the immobilization of Hb on the nanohybrid film of MWCNTs and gold colloidal nanoparticles [110]. A wide range of linear response from 0.21 μM to 3.0 mM with a detection limit of 80 nM was obtained. Tripathi *et al*. entrapped HRP in an ormosil composite doped with ferrocene monocarboxylic acid–bovine serum albumin conjugate and MWCNTs for a H_2_O_2_ biosensor [111]. MWCNTs improved the conductivity of the composite film and HRP provided a fast amperometric response to H_2_O_2_. A wide linear range between 20 μM and 4.0 mM with a detection limit of 5.0 μM (S/N = 3) was achieved. Luo *et al*. developed a H_2_O_2_ biosensor with an improved performance based on the immobilization of HRP onto electropolymerized PANI films doped with CNTs [112]. It was found that the existence of CNTs in the biosensor system could effectively increase the amount and stability of the immobilized HRP as well as the performance of the biosensor. A H_2_O_2_ biosensor based on the modification of graphite electrode with toluidine blue (Tb) modified MWCNTs was developed by H. Ju and co-workers [113]. HRP was immobilized with the aid of CS for sensing H_2_O_2_ with a good stability and reproducibility. Qian and Yang developed a mediator free amperometric biosensor for H_2_O_2_ based on composite film of MWCNTs/CS [114]. HRP was cross-linked with composite film using GA. Sanchez *et al*. reported the fabrication of MWCNTs/polysulfone biocomposite membrane, which allows the incorporation of HRP enzyme by phase inversion technique. This biocomposite membrane was used for the construction of a H_2_O_2_ biosensor [115]. Qu *et al*. took the combined advantages of CNTs and nano-Fe_3_O_4_ to prepare a magnetic CNTs/nano-Fe_3_O_4_ composite by co-precipitation. It exhibited higher electrocatalytic activity toward the redox processes of H_2_O_2_ [116]. They also introduced CS into the bulk of the composite by co-precipitation to immobilize GOx covalently to make an amperometric glucose sensor.

Choi *et al*. constructed a highly sensitive and stable amperometric ethanol biosensor based on the immobilization of ADH within a thin composite film of CNTs-titania-Nafion [117]. Due to the mesoporous nature of this composite film, the present ethanol biosensor exhibited remarkably fast response time and wide linear response range. Santos *et al*. constructed an amperometric ethanol biosensor based on co-immobilization of ADH and Methylene Blue (MB) on MWCNTs through the cross-linking with GA and agglutination with mineral oil [118]. The amperometric response of this biosensor showed an excellent operational stability and wide linear response range. Cai and co-workers fabricated a nanocomposite by the functionalization of SWCNTs with poly(Nile Blue A) for ethanol biosensor [119]. Immobilization of ADH onto the modified electrode showed electrocatalytic activity toward the oxidation of ethanol with a good stability, reproducibility, and higher biological affinity. Liu and Cai developed an ethanol biosensor based on the nanocomposites of positively charged PDDA wrapped SWCNTs [120]. The negatively charged ADH was immobilized on the nanocomposites via the charge interaction and this biosensor provided a good electrocatalytic activity toward the oxidation of ethanol with a good stability, reproducibility, and high biological affinity.

Yang *et al*. reported the modification of gold electrodes by self-assembling the positively charged Pt nanoparticle-MWCNTs-CS and negatively charged poly (sodium-*p*-styrenesulfonate) salt (PSS) for a sensitive cholesterol biosensor [121]. MWCNTs were dispersed in the Pt nanoparticle-doped CS solution to obtained Pt-CNTs-CHIT material. Cholesterol oxidase was immobilized onto the modified electrode surface using GA.

Tang *et al*. developed an amperometric glutamate biosensor based on self-assembly of glutamate dehydrogenase (GLDH) and poly (amidoamine) dendrimer-encapsulated platinum nanoparticles (Pt-PAMAM) onto carboxylic acid group-functionalized MWCNTs [122]. The modified electrode showed electrocatalytic activity toward the oxidation of glutamate with a good reproducibility and high sensitivity. Lin’s group fabricated a simple and inexpensive choline biosensor based on the immobilization of choline oxidase (ChO), ChO and HRP bienzymes onto MWCNTs modified GCE using LBL assembly technique [123]. With this configuration, a wide linear response range from 50 μM to 5.0 mM with a detection limit of about 10 μM for choline was achieved. Song *et al*. fabricated an another choline biosensor by immobilizing ChO into a sol-gel silicate film on MWCNTs modified platinum electrode [124]. This biosensor was used to detect choline released from lecithin by phospholipase D (PLD) in serum samples with high sensitivity and the detection limit was 0.1 μM. A stable and sensitive acetylthiocholine sensor based on immobilization of acetylcholinesterase (AChE) on the CS-MWCNTs composite was developed by Du *et al*. [125]. GA was used as cross linker to covalently bond AChE and efficiently prevent leakage of the enzyme from the film.

Lee *et al*. developed an amperometric tyrosinase biosensor based on MWCNTs dispersed in mesoporous composite films of sol-gel-derived titania and Nafion [126]. Tyrosinase was immobilized within the composite film and phenolic compounds were determined by the direct reduction of biocatalytically liberated quinone species. This sensor exhibited remarkably fast response time less than 3 sec and a good performance in terms of the sensitivity (417 mA/M) and the detection limit (0.95 nM) due to the large pore size of the composite film. An amperometric biosensor was based on the immobilization of HRP on MB modified MWCNTs for phenolic compounds and it showed a very wide linear response with a good sensitivity for catechol [127]. Recently, Lopez *et al*. described a biosensor fabricated by the modification of GCE with a matrix based on MWCNTs, tetrahydrofuran (THF) mixed with poly(vinylchloride) (PVC), and with a GA solution (MWCNTs-TPG/GC) for NADH detection [128]. The modified electrode showed a relatively higher sensitivity, a promotion of electron transfer, and it facilitated the amperometric determination of NADH starting in a potential of +0.40 V. Male *et al*. developed a biosensor for arsenite by depositing molybdenum-containing arsenite oxidase galvanostatically onto the active surface of a MWCNTs on GCE [129]. The detection limit of 1 ppb was found for arsenite but there was a severe interference caused by common metal ions found in tap and river waters. Mita *et al*. constructed a bisphenol A biosensor using a various tyrosinase containing CPE and they optimized the experimental condition with the composition of 10% tyrosinase, 45% SWCNTs, and 45% mineral oil [130]. It showed a good reproducibility with a detection limit of 20 nM. Liu and Lin fabricated an amperometric biosensor based on LBL self-assembling AChE on CNTs-modified GCE and integrated it within a flow injection-detection system. It was highly sensitive for organophosphate pesticides and nerve agents and showed a good precision, reproducibility, and stability [131]. CNTs play a dual significant role in this structure. It provides a robust immobilization sites for a suitable microenvironment to retain the enzyme activity and as a transducer, which amplifies the electrochemical signal of the product of the enzymatic reaction. Rahman *et al*. fabricated an amperometric lactate biosensor based on MWCNTs and conducting polymer (CP) by covalently immobilizing the lactate dehydrogenase and NADH onto the MWCNTs/CP assembly [132]. The MWCNTs/CP nanocomposite assembly was obtained through the electrochemical polymerization of monomer containing MWCNTs. The analytical results such as sensitivity, selectivity, and stability were found to be improved significantly using MWCNTs/ CP nanocomposite assembly.

**Figure 4.**
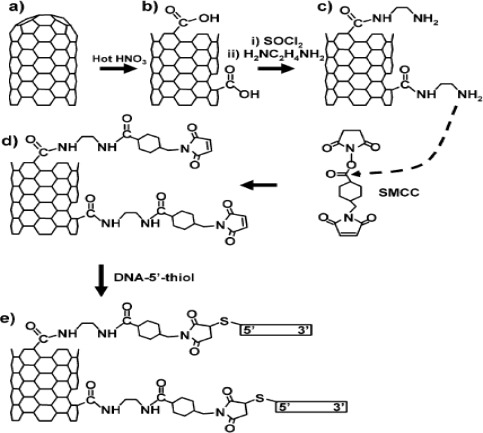
Overall scheme for fabrication of covalently linked DNA-nanotube adducts. Reprinted with permission from [134]. Copyright (2002) American Chemical Society.

The detection of DNA is currently an area of tremendous interest in genetics, clinics, pathology, criminology, pharmacogenetics, food safety, and many other fields. Most of DNA biosensors are developed based on the immobilization of single-stranded DNA onto the electrode surface labeled with an electrochemical indicator to recognize its complementary target sequence. CNTs are promising materials for the development of electrochemical DNA hybridization biosensors. The unique properties of CNTs can be united with the specific molecular-recognition features of DNA by coupling SWNTs to peptide nucleic acid and hybridizing these macromolecular wires with complementary DNA [133]. Both covalent and non-covalent linkage of DNA with CNTs have been reported where the former provide the best stability, accessibility, and selectivity during competitive hybridization [134]. Figure 4 shows an overview of the covalent attachment process. By this attachment, it was found that DNA molecules are accessible to hybridization and strongly favor hybridization with molecules having complementary sequences compared with noncomplementary sequences. The integration of CNTs with other materials has been also used for the immobilization of DNA. Yang *et al*. described a sensitive DNA hybridization biosensor based on ZrO_2_ nanoparticles and MWCNTs [135]. The MWCNTs/nano ZrO_2_/CS-modified GCE was fabricated by dispersing ZrO_2_ nanoparticles and MWCNTs in CS and oligonucleotides were immobilized on a modified GCE. The hybridization reaction on the electrode was monitored by DPV analysis where electroactive daunomycin was used as an indicator. Jiao and co-workers applied the same approach for DNA biosensor using ZnO nanoparticles instead of ZrO_2_ nanoparticles [136]. Recently, Ma *et al*. fabricated an electrochemical DNA biosensor based on LBL self-assembly of MWCNTs and GNPs via covalent-bonding interaction [137]. Doxorubicin was used as an intercalator and the hybridization events were monitored electrochemically by DPV measurement. The biosensor showed an improved sensitivity with an excellent reproducibility due to the high catalytic activities of GNPs and the ability of CNTs to promote electron-transfer reactions. A wide linear response range from 0.5 to 0.01 nM with a detection limit of 7.5 pM for target DNA was achieved. Recently, Niu *et al*. used manganese complex of rutin as a redox intercalator with carboxylic acid group-functionalized MWCNTs and fabricated DNA biosensor for DNA hybridization detection [138]. The modified electrode dramatically increased the amount of DNA attachment and the sensitivity of the complementary ssDNA detection mostly due to the large surface area and good charge-transport characteristics of CNTs. Erdem *et al*. described a new DNA biosensor based on the enhancement of guanine signal at MWCNTs-modified pencil graphite electrode (PGE) using DPV [139]. PGE behaved as a microelectrode array coupled with its higher porosity and showed improved performance compared to GCE. Another new DNA biosensor based on electrochemical impedance was described by Fang’s group [140]. They modified GCE using a composite material of PPy and carboxylic group-terminated MWCNTs. A probe with an amino group-termination was linked onto the PPy/MWCNTs-COOH/GCE by using EDAC and it was found that the hybridization reaction with its complementary decreased the electron transfer resistance. Zhang’s group also used PPy and MWCNTs to develop a high sensitive and selective biosensor for DNA hybridization based on the immobilization of DNA probe within electropolymerized PPy on a MWCNTs paste electrode [141]. Ethidium bromide (EB) was used as an intercalator and the current change generated from it was monitored. Only the complementary DNA, compared to the five-point mismatched and non-complementary sequences, gave an obvious current flow and a detection limit of 0.85 pM was obtained. An electrochemical DNA biosensor based on palladium nanoparticles combined with MWCNTs was suggested by Chang *et al*. [142]. MB was used as an indicator and the hybridization was monitored by DPV measurement. A lower detection limit of 120 fM for the target DNA was achieved and the improved sensitivity was attributed to the ability of CNTs promoting electron-transfer process and the high catalytic activities of palladium nanoparticles for electrochemical reaction of MB.

**Figure 5.**
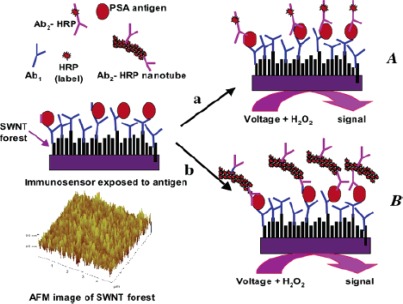
Configuration of the nanotube immunosensor. Reprinted with permission from [145]. Copyright (2006) American Chemical Society.

Electrochemical immunosensors are the popular area of study due to their high specificity, sensitivity, and stability. Most of approaches are based on an enzyme-linked immunosorbent assay (ELISA) system built on electrode surfaces. The amount of enzyme-linked antigen bound to the immobilized antibody is determined by the relative concentration of the free and conjugated antigen and quantified by the rate of enzymic reaction. To avoid the additional steps for labeling, the label-free immunosensors are getting much interest. CNTs are potential materials for the fabrication of labeled or label-free immunosensors. The carboxylic acid groups-functionalized CNTs can be used as an antibody immobilizing platform. CNTs can also be used in the detection probe of an immunosensor. Yun *et al*. developed a label-free immunosensor based on CNTs array electrodes for direct electrochemical detection of antigen–antibody binding reaction [143]. Anti-mouse IgG was covalently immobilized on the carboxylic acid-terminated nanotube array and the binding was characterized by CV and electrochemical impedance spectroscopy (EIS). The detection limit was found to be 200 ng/mL. EIS was chosen here as the analytical approach because of its ability to analyze the electrochemical response of the electrode over a wide frequency range. Electrodes modified with SWCNTs array have also been used by Okuno *et al*. to fabricate a label-free electrochemical immunosensor for prostate-specific antigen [144]. Yu *et al*. described an electrochemical immunosensor using SWCNTs forest platforms with multi-label secondary antibody-nanotube bioconjugates for highly sensitive detection of a cancer biomarker in serum and tissue lysates [145]. A great amplification of the sensitivity was achieved by using bioconjugates featuring HRP labels and secondary antibodies (Ab2) linked to MWCNTs at high HRP/Ab2 ratio (Figure 5). A low detection limit of 4 pg/mL for prostate specific antigen was obtained in 10 μL undiluted calf serum with this strategy.

Polymer-CNTs composite films have been widely used for the fabrication of electrochemical immunosensors. Cataldo *et al*. evaluated an amperometric immunosensor based on the covalently bound anti-biotin antibodies (Ab) embedded into a polylysine (PLL)-SWCNTs composite layer [146]. An improved amperometric detection limit of 10 pM was obtained for biotin (Ag) labeled with HRP by incorporating SWCNTs into PLL-antibody assemblies. One advantage of this proposed immunosensor is the improved thermal stability of the anti-biotin embedded into the PLL matrix. Poly (3,4-ethylene-dioxythiophene)-coated MWCNTs have been used for the first time as a voltammetric sensor in an immunoassay for Cholera Toxin (CT) by Ho’s group [147]. Ganglioside (GM1)-functionalized liposomes encapsulated with potassium ferrocyanide (an electroactive redox marker) were used for the detection of CT. A wide linear range from 10 fg/mL to 0.1 μg/mL with a detection limit of 0.1 fg/mL of CT was achieved. Sánchez *et al*. described an electrochemical immunosensor method based on polysulfone membrane encapsulating MWCNTs and immunoreagents layered on disposable screen-printed electrodes [148]. The immunocomposite acts both as reservoir of immunological material and transducer while offering high surface area, high toughness, and mechanical flexibility.

Chitosan (CS), a biological cationic macromolecule, provides a suitable environment for biomolecules to keep their activity. Their combination with highly conductive CNTs as an immunosensing platform has attracted considerable interest. Recently, Li *et al*. proposed a novel amperometric immunosensor for human chorionic gonadotropin (HCG) assay incorporating Tb and Hb on the MWCNTs–CS modified GCE, followed by electrostatic adsorption of a conducting GNPs film as sensing interface [149]. The MWCNTs-CS matrix provided a congenial microenvironment for the immobilization of biomolecules and promoted the electron transfer of the redox active species, thus, enhanced the sensitivity of the immunosensor. CS can be also used for the development of a highly sensitive and label-free amperometric immunosensor for carcinoembryonic antigen detection [150]. The fabrication of the immunosensor was based on LBL assembly of GNPs, MWCNTs-thionine, and CS on 3-mercaptopropanesulfonic acid (MPS)-modified gold electrode surface. The detection limit of carcinoembryonic antigen with this label-free amperometric immunosensor was determined to be 0.01 ng/ml.

The practical application of CNTs can be found in a very limited area only. It is partially attributable to the lack of simple modification skill of electrode surfaces with CNTs. To overcome this obstacle, Yang and coworkers developed two different methods for the modification of ITO electrode with CNTs. The ITO electrodes are advantageous to use in optical experiment and to make electrochemical (bio) sensors because it is optically transparent and display high conductivity with low background current. Initially, they fabricated thin film of MWCNTs by simply physisorbing of CNTs on the ITO electrode from the aqueous solution of carboxylated MWCNTs [151]. To make thin film of CNTs on ITO electrode, they covalently immobilized the carboxylated SWCNTs on an amine-functionalized ITO surface using dicyclohexylcarbodiimide as a coupling agent [152]. Both films showed low background currents and good electrocatalytic properties toward the oxidation of *p*-aminophenol (*p*-AP), which is an enzymatic product of enzyme substrate (*p*-aminophenyl phosphate). Both thin CNT films could be used to fabricate sandwich-type immunosensors because of the good electrocatlytic property with a low background current, and the ability of immobilization of biomolecules on the sidewall of the CNTs. To fabricate the sandwich type immunosensor, avidin was immobilized first on the hydrophobic sidewalls of CNTs to capture biotinylated anti-mouse IgG. Then, the target mouse IgG was sandwiched between the biotinylated anti-mouse IgG and alkaline phosphatase-conjugated antimouse IgG. The alkaline phosphatase catalyzed the conversion of electroactive *p*-AP from non electroactive *p*-aminophenyl phosphate. The enhancement of the generation of *p*-AP upon increase of the concentration of target mouse IgG resulted in the strength of the signal. The thin films of MWCNTs and SWCNTs gave the detection limits of 10 pg/mL and 100 pg/mL, respectively. They are comparable to those of sensors with a commonly employed enzyme-linked immunosorbent assay (pM range).

## 5. Application of CNTs-Based Sensors to Real Sample Analysis

CNTs-based sensors can be applied in real sample analysis in different areas such as biomedical, food, agriculture, and fishing industries. There are many biomedical sensing applications where CNTs-based sensors perform better in real sample analysis. CNTs-based sensors can be used in commercial food samples to detect undesired chemical residues resulting from animal drugs, food additives, pesticides, and other environmental contaminants in raw and processed foods. Rahman *et al*. determined the lactate concentration in commercial milk using CNTs-based electrochemical biosensor and compared the result with that obtained from a biochemical analyzer [132]. The biosensor result was found to be in good agreement with the result from biochemical analyzer (0.18 ± 0.006 mM and 0.174 ± 0.01 mM, respectively). CNTs based electrochemical sensors are also widely used in real blood and urine samples analyses. Table 1 summarizes the applications of some CNTs-based electrochemical sensors to real sample analyses.

## 6. Conclusions

CNTs are now used extensively in the fabrication of novel nanostructured electrochemical sensors. CNTs-modified electrodes have many advantages over other forms of carbon electrodes due to their small size, high electrical and thermal conductivity, high chemical stability, high mechanical strength, and high specific surface area. Their small diameter and long length allow them to be plugged into proteins with better electro-activity compared to other carbon based electrodes. The promoted electron transfer and direct electrochemistry of proteins at CNTs-based electrochemical sensing films are now well documented. Due to its faster electron transfer over other carbon based materials, CNTs show excellent electrocatalytic activity in redox behavior of different compounds. Analytical sensing at CNTs-modified electrodes results in low detection limits, high sensitivities, reduction of overpotentials, and resistance to surface fouling. The aforementioned outstanding properties of CNTs make them an exciting alternative for the development of novel electrochemical sensors and biosensors. However, there are a number of challenges to be addressed to fulfill the application of CNTs for sensors. The commercial production of pure and defect-free CNTs is difficult and costly. Processing of CNTs is still not fully controlled. For example, aggregations of tubes are not prevented, lengths are not uniformly obtainable, and non-specific adsorptions of proteins to the walls of nanotubes are not prevented. CNTs can cause health risks due to their toxicity and harmful effects in the lung, where they can agglomerate leading to suffocation. The toxicological impact of nanotubes is an obstacle for the application of nanotubes in bioelectronics and any subject integrated with living biological systems. Another limitation is that CNTs are commonly insoluble in most solvents, which has greatly hindered their promising practical applications. Covalent and non-covalent functionalizations of CNTs are not very effective in overcoming this limitation. CNTs can be dispersed in Nafion, Teflon, CS, mineral oils, sol-gel silica, and in some polymer but these methods possibly impair the chemical properties of CNTs or decrease their conductivity. While there have some disadvantages of CNT-modified electrode for sensing application, the continuous growing research interest in this field is contributing to overcome them. It is believed that the merits of CNT-based sensors will bring dramatic changes to future sensor industry.

## Figures and Tables

**Table 1.** Applications of CNTs-based electrochemical sensors in real samples.

**Electrode**	**Analyte**	**Real sample**	**Detection limit**	**Reference**
GCE/P3MT/SWNTs/Nafion	dopamine	serum	5.00 nM	[23]
GCE/ PANI /MWNTs /β-CD	dopamine	injection	12.0 nM	[25]
Au/SWNTs	rutin	tablet (drug)	10.0 nM	[54]
GCE/ MWNTs /β-CD	rutin	urine	0.20 μM	[55]
GCE/ MWNTs / PtNC	estrogen	serum	0.18 μM	[60]
CPE/ MWCNTs/CoSal	tryptophan	serum	0.10 μM	[64]
GCE /SWNTs /BMIMPF_6_	methylparathion	lake water /apple	1.00 nM	[67]
GCE /SWNTs /BMIMPF_6_	xanthine	serum / urine	2.00 nM	[68]
GCE/ MWNTs / poly-ACBK	dihydroxybenzene	water	0.10 μM	[73]
GCE/ MWNTs	noscapine	drug/blood	80.0 nM	[79]
GCE/ MWNTs	captopril	drug/urine	0.20 μM	[80]
ITO/ MWCNTs/GOx/NFE	glucose	serum	1.00 μM	[96]
Teflon/MWCNTs/Au_coll_/GOx	glucose	beverage	17.0 μM	[98]
Teflon/MWCNTs/Au_col_/ADH	ethanol	beer	4.70 μM	[99]
GCE/CS/CNTs/ Au–PtNPs	glucose	blood/urine	0.20 μM	[103]
GCE/MWNTs/ FMC–BSA	hydrogen peroxide	milk	0.20 μM	[111]
CPE/MB/MWCNTs/ADH	ethanol	beverage	5.00 μM	[118]
Pt/MWCNTs/ChOx	choline	serum	0.10 μM	[124]
Au/pTTCA/MWNTs/LDH	lactate	milk/serum	1.00 μM	[132]

P3MT, poly(3-methylthiophene); PANI, polyaniline; β-CD, β-cyclodextrin; ABSA, aminobenzene sulphonic acid; PtNC, Platinum nano-clusters; CPE, carbon paste electrode; CoSal, cobalt salophen; BMIMPF_6,_ 1-butyl-3-methylimidazolium hexafluophosphate; ACBK, acid chrome blue K; ADH, alcohol dehydrogenase; CS, chitosan; FMC–BSA, ferrocene monocarboxylic acid–bovine serum albumin; ChOx, choline oxidase; pTTCA, poly-5,2′-5′,2″-terthiophene-3′-carboxylic acid; LDH, Lactate dehydrogenase
